# QSAR-based physiologically based pharmacokinetic (PBPK) modeling for 34 fentanyl analogs: model validation, human pharmacokinetic prediction and abuse risk insights

**DOI:** 10.3389/fphar.2025.1692293

**Published:** 2025-10-17

**Authors:** Simeng Zhang, Yawen Xu, Xianbin Zeng, Jingzhi Ran, Yuanyuan Chen, Lixin Kuai, Kaixi Li, Peng Xu, Fang Yan, Dan Wang

**Affiliations:** ^1^ School of Pharmacy, China Pharmacokinetic University, Nanjing, China; ^2^ Office of China National Narcotics Control Commission, China Pharmacokinetic University Joint Laboratory on Key Technologies of Narcotics Control, Beijing, China; ^3^ Beijing Narcotics Control Technology Center, Beijing, China; ^4^ Key Laboratory of Drug Monitoring and Control, Drug Intelligence and Forensic Center, Ministry of Public Security, Beijing, China

**Keywords:** fentanyl analogs, PBPK model, human pharmacokinetics, QSAR model, abuse risk

## Abstract

**Introduction:**

Fentanyl analogs, as emerging new psychoactive substances (NPS), pose a global public health threat due to widespread abuse, high toxicity, and frequent overdose fatalities. However, their structural diversity and scarce experimental pharmacokinetic (PK) data hinder hazard and abuse risk assessment. Conventional physiologically based pharmacokinetic (PBPK) models for these analogs are limited by reliance on time-consuming *in vitro* experiments or error-prone interspecies extrapolation for key parameters (e.g., tissue/blood partition coefficient, Kp).

**Methods:**

To address this, we developed and validated a QSAR-integrated PBPK framework (QSAR: Quantitative Structure-Activity Relationship) for predicting human PK of fentanyl analogs. The workflow included: (1) Validating the framework *via* intravenous β-hydroxythiofentanyl in Sprague-Dawley rats (QSAR-predicted Kp *via* Lukacova method, GastroPlus^®^ modeling); (2) Comparing Kp accuracy (literature *in vitro* data, QSAR, interspecies extrapolation) in rat/human fentanyl PBPK models; (3) Predicting PK and tissue distribution (plasma +10 organs including brain/heart) of 34 human fentanyl analogs.

**Results:**

Key results: (1) For β-hydroxythiofentanyl, all predicted rat PK parameters (area under the plasma concentration-time curve from time zero to the last measurable time point [AUC_0-t_], teady-state volume of distribution [V_ss_], and elimination half-life [T_1/2_]) of rats fell within a 2-fold range of the experimental values; (2) In human fentanyl models, QSAR-predicted Kp improved accuracy (V_ss_ error: >3-fold [extrapolation] vs. <1.5-fold [QSAR]) (3) Among 34 analogs, eight (e.g., p-fluorofentanyl); had brain/plasma ratio >1.2 (vs. fentanyl’s 1.0), indicating higher CNS penetration and abuse risk.

**Discussion:**

This study demonstrates that the QSAR-PBPK framework enables rapid prediction of human pharmacokinetics (PK) for understudied fentanyl analogs without relying on scarce experimental data. For structurally similar, clinically characterized analogs (e.g., sufentanil, alfentanil), predictions of key PK parameters (e.g., T_1/2_, V_ss_) fall within 1.3–1.7-fold of clinical data, supporting the framework’s utility for generating testable hypotheses about the PK of understudied analogs. It not only fills the data gap for fentanyl analog hazard assessment but also provides a scalable modeling strategy for PK evaluation of other NPS or illicit drugs.

## 1 Introduction

Fentanyl, a potent synthetic opioid receptor agonist, was initially developed to exert robust analgesic effects. Clinically, pharmaceutical fentanyl is widely employed in the management of various pain conditions, post-operative analgesia, and as an adjunct to anesthetics during surgical procedures (Patocka et al., 2024). Alarmingly, the 2024 World Drug Report highlights that fatalities attributed to fentanyl overdoses in North America surged to unprecedented levels during the COVID-19 pandemic (UNODC, 2024). Compounding this challenge, the relative ease of synthesis and structural modification of fentanyl has led to the proliferation of numerous analogs with diverse biological activities. Unfortunately, the pace of research has failed to keep abreast of the emergence and rapid spread of these new fentanyl analogs. For example, after Sweden classified acetylfentanyl and butyrfentanyl as controlled substances and implemented bans, these derivatives disappeared from online marketplaces, only to be replaced by variants such as furanylfentanyl and 4-methoxybutyrfentanyl (Helander et al., 2017). Consequently, a rapid understanding of the pharmacokinetic profiles of emerging analogs is of paramount importance for formulating relevant therapeutic strategies and establishing effective regulatory policies.​

Physiologically based pharmacokinetic (PBPK) models represent advanced computational tools that predict the concentration-time profiles of compounds in plasma across different species. This is accomplished by integrating the physicochemical properties of the compound with the physiological characteristics of the target species (Sager et al., 2015). Key parameters such as logD, pKa, and the unbound fraction to plasma proteins (Fup) can be obtained either through *in vitro* measurements or predicted using quantitative structure-activity relationship (QSAR) models (Miller et al., 2019). Moreover, by leveraging these input parameters, PBPK models can predict chemical concentration changes in various tissues, enabling the rapid screening of substances with a quick onset of action and facilitating the elucidation of the pharmacological properties of specific compounds.​

However, traditional methods for evaluating model performance rely on pharmacokinetic (PK) data specific to the target chemical, rendering them inadequate for fentanyl derivatives that have not been sufficiently studied (EPA, 2006). Previous research has explored the hypothesis that PBPK models fully developed for a target chemical (with unavailable PK data) can be evaluated using PK data from its structural or functional analogs (with available PK data) (Ellison, 2018). Fentanyl, being extensively documented in clinical literature, provides a rich dataset that can be used to assess the accuracy of models for fentanyl analogs.

Notably, our approach constitutes a notable advancement in the field of PBPK modeling for fentanyl analogs compared with prior investigations. Earlier studies were often constrained by two key limitations: they either relied exclusively on *in vitro* assays for parameterization—a process inherently characterized by high time and resource consumption—or restricted the application of QSAR predictions to the estimation of opioid-opioid receptor affinity (Floresta et al., 2019), rather than leveraging QSAR for the prediction of PBPK-essential critical parameters (e.g., logD, pKa, plasma protein unbound fraction). In distinct contrast, our work integrates the predictive capacity of QSAR with PBPK modeling frameworks. Through a systematic comparative analysis of parameters derived from *in vitro* measurements *versus* QSAR predictions, we successfully identified the optimal parameter source, which directly contributes to enhanced model accuracy.​ Furthermore, the QSAR integration in our study represents a significant improvement over existing models. By harnessing QSAR to predict PBPK-essential parameters, we reduce reliance on scarce *in vitro* data and accelerate the modeling process. This not only addresses the data gap for understudied fentanyl analogs but also enhances the speed of pharmacokinetic prediction without compromising accuracy—a claim validated by comparisons with measured experimental data.

**FIGURE 1:**
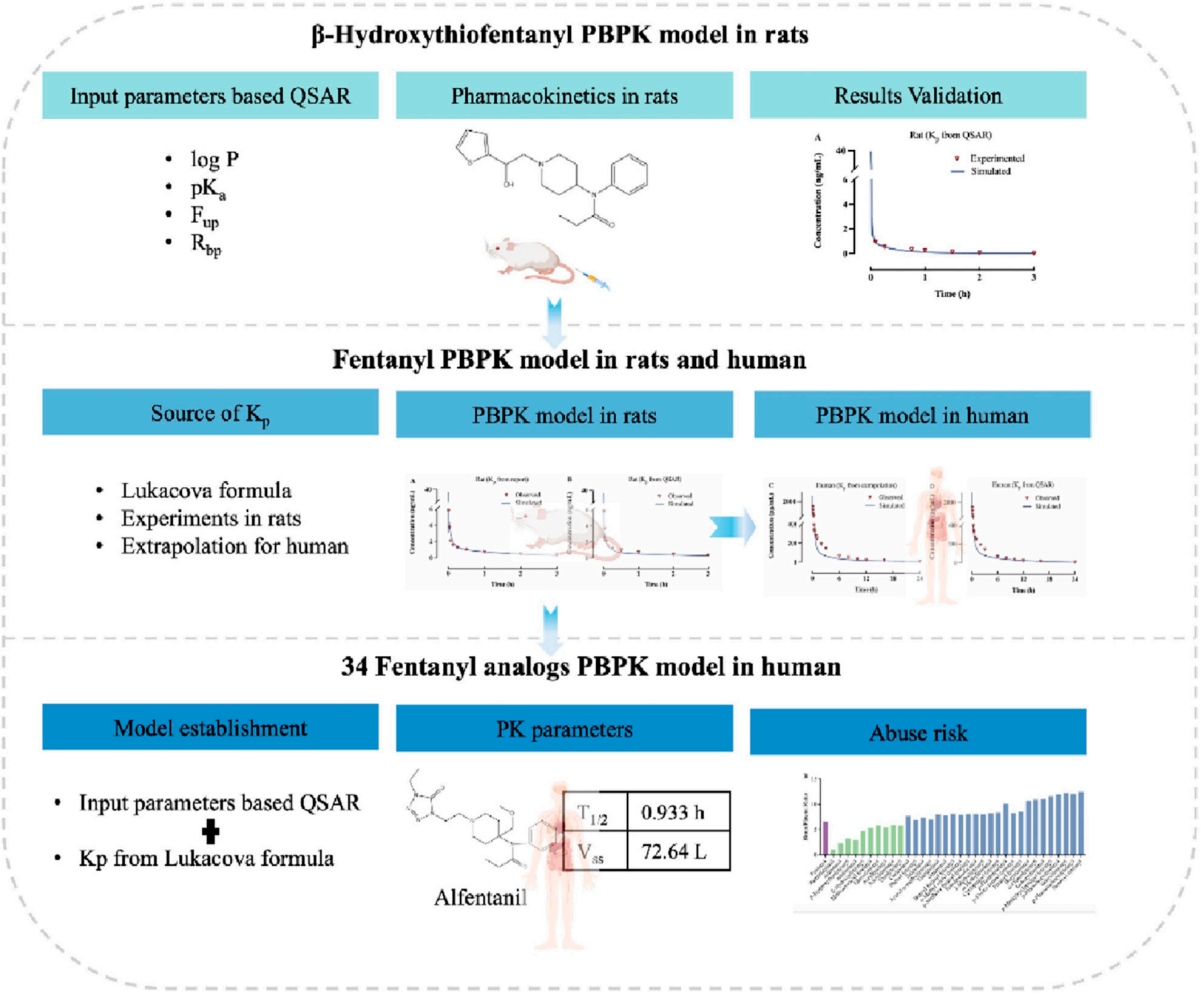
Experimental flowchart for predicting human ADME/PK of fentanyl analogs using PBPK modeling.

The objectives of this study are threefold: 1) to evaluate the accuracy of the PBPK model based on QSAR-predicted data using measured PK data of beta-hydroxythiofentanyl in rats; 2) to develop PBPK models for fentanyl in both rats and humans, with modeling parameters derived from *in vitro* measurements and QSAR predictions respectively, and to compare the accuracy of these parameters during the modeling process; 3) to predict the pharmacokinetics of 34 fentanyl analogs in human plasma and various tissues, thereby bridging the existing data gap in this field. The study design and workflow are illustrated in Figure 1.

## 2 Materials and methods

### 2.1 Materials

#### 2.1.1 In silico programs

Fentanyl and its derivatives were identified through a comprehensive search of peer-reviewed journal publications (PubMed, National Library of Medicine). The structural formulas of fentanyl analogs were obtained from the PubChem Website based on the compound name (https://pubchem.ncbi.nlm.nih.gov). QSAR models predicted the physiochemical and pharmacokinetic properties of fentanyl analogs in ADMET Predictor v. 10.4.0.0 (AP, Simulations Plus, Inc.). PBPK modeling and simulations were established using GastroPlus v. 9.8.3 (GP, Simulations Plus, Inc. Lancaster, CA). The PK parameters were estimated using Phoenix WinNonlin software (version 8.3).

#### 2.1.2 Animals

Male Sprague-Dawley (SD) rats, aged 6–8°weeks old, were procured from SPF Biotechnology Co.,Ltd (Beijing, China). The animals were housed in a controlled environment with humidity maintained at 50% ± 10% and temperature at 25 C ± 2 C, under a 12 h light/dark cycle They had *ad libitum* access to food and water. All experiments were conducted according to protocols approved by the Institutional Welfare and Ethics Committee for Laboratory Animals, Key Laboratory of Drug Monitoring and Control.

#### 2.1.3 Chemicals and reagents

Beta-hydroxythiofentanyl (content ≥98%) was provided by the Drug Intelligence and Forensic Center of the Ministry of Public Security, China. 0.9% saline solution was obtained from Shandong Qidu Pharmaceutical Co., Ltd. Acetonitrile and formic acid were sourced from Sigma-Aldrich (Saint Louis, MO). All other reagents utilized in the experiment were commercially available and of analytical or HPLC grade. The triple quadrupole mass spectrometric detector (6,500+; AB SCIEX Technologies) was used for LC-MS/MS analysis.

### 2.2 Methods

#### 2.2.1 Development and validation of the β-hydroxythiofentanyl PBPK model in rats

To assess the modeling approach based on QSAR predictions, our research developed the PBPK model for intravenous β-hydroxythiofentanyl in rats and compared the experimentally obtained concentration and time profiles and pharmacokinetic parameters with the predicted data.

Time points for the study were 0 min, 15 min, 45 min, 60 min, 90 min, 120 min, 180 min, and 240 min post-dosing with 7 μg/kg iv. dose of β-hydroxythiofentanyl. For the pharmacokinetic assessment, 400 μL of blood was collected and subsequently centrifuged at 4,000 rpm for 5 min. The plasma was then carefully transferred into microcentrifuge tubes and stored in a freezer at −20 °C until further analysis. On the day of testing, the plasma samples were analyzed using LC-MS. Non-compartmental analyses were conducted utilizing Phoenix WinNonlin software to estimate pharmacokinetic parameters.

For the PBPK model of β-hydroxythiofentanyl constructed using QSAR predictions, the molecular structure of β-hydroxythiofentanyl was input as the core descriptor. The tissue/blood partition coefficient (Kp) shown in Table 1, a critical parameter governing tissue distribution in PBPK modeling, was predicted using the Lukacova method. It was a structure-driven QSAR approach widely applied for estimating tissue partition coefficients of small-molecule compounds based on their structural features. The QSAR-predicted Kp values were incorporated into GastroPlus^®^ software to construct the PBPK model for β-hydroxythiofentanyl. For β-hydroxythiofentanyl, the systemic clearance (CL_sys_), a critical parameter for PBPK model parameterization, was derived directly from *in vivo* experimental data, rather than QSAR prediction. The CL_sys_ value obtained in the β-hydroxythiofentanyl model prediction section is derived from the CL_sys_ value predicted by GastroPlus^®^ software after inputting the experimentally measured CL_sys_ value and the QSAR-predicted Kp value.

**TABLE 1:** The tissue/blood partition coefficient (Kp) for β-hydroxythiofentanyl and fentanyl in rats and humans.

Kp: The tissue/blood partition coefficient	Lung	Adipose	Muscle	Liver	Spleen	Heart	Brain	Kidney	Skin	Reproductive organs	Red marrow	Yellow marrow	Rest of body
Kp of β-hydroxythiofentanyl in rats based on QSAR	3.72	1.62	1.98	4.91	3.42	2.66	1.73	5.03	1.92	5.03	2.03	1.62	3.42
Kp of fentanyl in rats from report	15	30	3.5	4.3	31	5.1	4	12	4.17	8.78	3.15	8.87	5.49
Kp of fentanyl in rats based on QSAR	13.90	17.63	5.96	15.02	9.92	8.46	32.48	15.88	7.88	15.88	6.06	17.63	9.92
Kp of fentanyl in humans based on interspecies extrapolation	12.56	25.11	2.93	3.6	25.95	4.27	3.35	10.04	3.49	7.35	2.64	7.42	4.60
Kp of fentanyl in humans based on QSAR	3.29	7.74	3.10	6.46	4.28	3.79	5.37	5.56	3.36	5.56	5.99	7.74	4.28

Finally, to validate the accuracy of this QSAR-based PBPK model, the model-predicted pharmacokinetic (PK) profiles of β-hydroxythiofentanyl were systematically compared with experimentally measured PK data of the compound. This comparative analysis allowed quantification of the agreement between predicted and observed PK outcomes, thereby verifying whether the QSAR-based parameterization (Lukacova method-derived Kp) could support reliable PBPK modeling of β-hydroxythiofentanyl.

#### 2.2.2 Development and validation of the fentanyl PBPK model in rats and humans

PBPK models for fentanyl were established to characterize the pharmacokinetic (PK) profiles of fentanyl following intravenous (IV) administration in rats and humans, respectively. In PBPK modeling, the Kp is recognized as a critical parameter for accurate prediction of tissue concentrations, as it directly governs the distribution of fentanyl between systemic circulation and peripheral tissues. Conventionally, Kp values are determined *via* multiple approaches, including *in vivo* animal PK studies, *in vitro* equilibrium dialysis (for measuring plasma protein binding and tissue affinity), and QSAR modeling (Yau et al., 2020). For the rat PBPK model, Kp values were initially derived from previously published literature data to ensure consistency with established experimental findings (Björkman et al., 1990). To further evaluate the reliability of QSAR-predicted Kp for model parameterization, an additional rat PBPK model was constructed using Kp values predicted via the Lukacova method—a structure-based QSAR approach that estimates tissue partition coefficients based on the molecular structure of the compound. This dual-parameter-source design allowed direct comparison of model performance between literature-derived and QSAR-predicted Kp.

A major challenge in studying fentanyl and its analogs is the scarcity of clinical PK data, as direct human studies are often constrained by ethical and safety considerations. To address this gap, PBPK models enable interspecies extrapolation of PK data from animal models (e.g., rats) to predict human absorption, distribution, metabolism, and excretion (ADME) profiles. In the present study, two distinct human fentanyl PBPK models were developed, differing only in their Kp parameter sources: QSAR-predicted Kp: Human Kp values were generated via the same Lukacova QSAR method applied to the rat model; Human Kp values were predicted from rat Kp values using the following formula:

$$Kp\left(human\right)=\frac{fup\left(human\right)}{fup\left(rat\right)}\times \frac{BP\left(rat\right)}{BP\left(human\right)}\times Kp\left(rat\right)$$

Physicochemical properties of fentanyl (logP, pKa, and solubility profiles) were predicted by GastroPlus software [fup (rat) = 8.3, fup (human) = 25.5, BP(rat) = 1.01, BP(human) = 1.01]. The human PBPK model was administered at a dose of 0.1 mg at 70 kg.

For the CL_sys_ value of fentanyl, the observed value was obtained from experimentally measured data reported in the literature. Subsequently, the experimentally measured CL_sys_ values corresponding to rats and humans, along with the Kp values derived from QSAR (Quantitative Structure-Activity Relationship) predictions and interspecies extrapolation, were separately input into the model for prediction. The corresponding predicted CL_sys_ values were then generated as outputs. The performance of the models was evaluated by comparing the predicted plasma concentration-time (C-t) profiles with experimental clinical data (for humans) and literature-derived PK data (for rats). The primary objective of this validation was to verify whether QSAR-based Kp parameterization yields a human PBPK model with higher predictive accuracy than the model relying on interspecies extrapolation of Kp (Lim et al., 2012, YK. et al., 2024).

#### 2.2.3 Development and application of QSAR-Based PBPK models for 34 fentanyl analogs in humans

Leveraging the established QSAR-based PBPK modeling framework, PBPK models were constructed for 34 fentanyl analogs to characterize their disposition following intravenous administration in humans.

The model development workflow was standardized across all analogs: First, QSAR modeling was employed to generate two core sets of input parameters: (1) tissue/blood partition coefficients (Kp) for each analog in human, predicted via the Lukacova method (consistent with the fentanyl model); and (2) key physicochemical properties of the analogs (e.g., logP, aqueous solubility), predicted using ADMET Predictor™. These QSAR-derived parameters were then integrated into the human PBPK modeling platform (GastroPlus^®^ software) to construct species-specific PBPK models for each fentanyl analog. It is noteworthy that the predictions for 34 fentanyl analogs in this study all utilized the experimentally measured CL_sys_ value of fentanyl in humans, as reported in the literature. This approach was adopted to achieve the effect of rapidly predicting the relevant data of this class of substances based on one analog. After inputting fentanyl’s CL_sys_ value and other parameters into the model, the predicted CL_sys_ values adjusted by the model can be generated as outputs.

This modeling subsequently enabled the prediction of their concentration and time profiles in plasma and ten tissues and organs, including the brain, heart, and adipose. Finally, the predictive capability of the model was evaluated using clinical data pertaining to fentanyl.

## 3 Results

### 3.1 QSAR-based PBPK results show good agreement with experimental measurements by β-hydroxythiofentanyl

**FIGURE 2:**
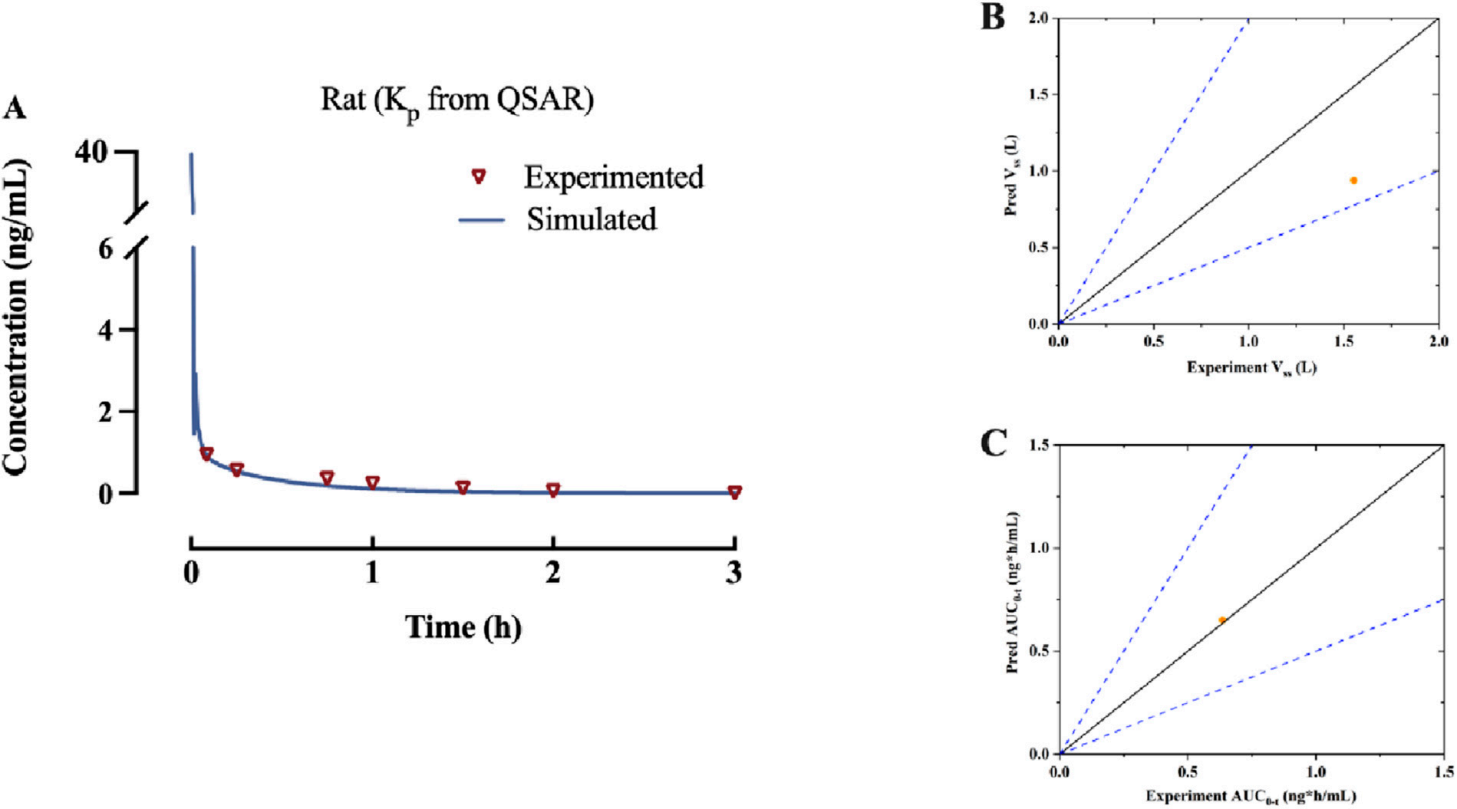
**(A)** Predicted and experimented concentration-time profiles of β-hydroxythiofentanyl following intravenous administration. **(B,C)** The experimented *versus* predicted graphs for pharmacokinetic parameters of β-hydroxythiofentanyl, including V_ss_ and AUC_0-t_. The orange dot represents data of K_p_ from QSAR.

According to the findings presented in 3.1, a PBPK model was developed for rats that were administered β-hydroxythiofentanyl via intravenous injection, using K_p_ predicted through QSAR. The data obtained from the experiment *versus* predicted plasma concentration and time profiles after intravenous administration are plotted in Figure 2. Based on visual inspection, the intravenous administration of β-hydroxythiofentanyl demonstrated great agreement between the data from experiment and the predicted. The model parameters and pharmacokinetic parameters from experimented and predicted are listed Table 2. The experimented and predicted values for V_ss_ and AUC_0-t_ are compared and illustrated in Figure 2. The predicted values for all pharmacokinetic parameters fell within a 2-fold error margin of the experimented values.

**TABLE 2:** The model parameters and pharmacokinetic parameters from experimented and predicted of β-hydroxythiofentanyl.

Species	Dose (μg)	Gender	Age		Weight (kg)	Log P		S_w_ (mg/mL)		F_up_	R_bp_		P_eff_ (cm^a^s^-1^)
Rats	1.75	male	6–8 weeks		0.25	2.59		0.48		32.25%	1.04		3.35^a^10^−4^

	CL_sys_ (L/h)			V_ss_ (L)			T^1/2^ (h)		AUC_0-t_ (ng·h/mL)			AUC_0-inf_ (ng·h/mL)	
Experimented	2.660			1.496			0.4625		0.635			0.669	
Predicted	2.687			0.938			0.242		0.650			0.651	

^a^ S_w_, water solubility; R_bp_, blood to plasma concentration ratio; P_eff_, effective human jejunal permeability.

### 3.2 QSAR-based PBPK results in human exhibit higher accuracy than those from interspecies extrapolation in human PBPK model for fentanyl

The PBPK model of fentanyl was respectively developed in rats and humans, with the injectable dose derived from the commonly utilized analgesic dosage of fentanyl in humans, which is 0.1 mg. This human dosage was converted to a corresponding amount based on body surface area, resulting in an administration of 1.75 μg to the rat subjects (Nair and Jacob, 2016). Fentanyl possesses a low molecular weight and high lipid solubility, which facilitate its diffusion across cellular membranes. Consequently, the model was developed using the perfusion rate-limited tissue model approach.

**FIGURE 3:**
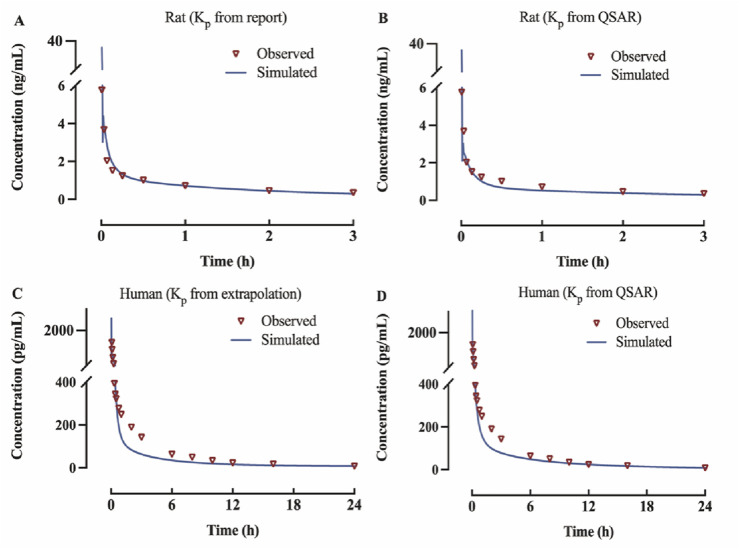
Predicted and observed concentration-time profiles of fentanyl following intravenous administration **(A)** rat model of K_p_ from report; **(B)** rat model of K_p_ from QSAR prediction; **(C)** human model of K_p_ from interspecies extrapolation; **(D)** human model of K_p_ from QSAR prediction.

**FIGURE 4:**
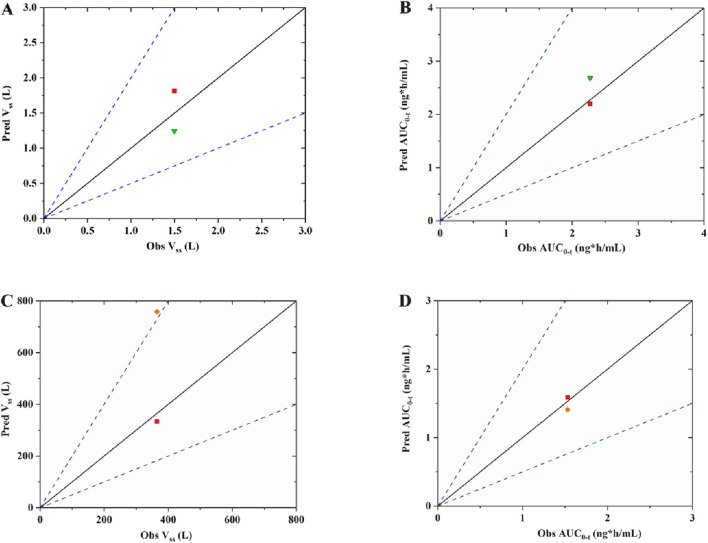
The observed *versus* predicted graphs for pharmacokinetic parameters of fentanyl **(A)** V_ss_ of rat model; **(B)** area under the plasma concentration-time curve from time zero to time t, AUC_0-t_ of rat mode; **(C)** V_ss_ of human model; **(D)** AUC_0-t_ of human model. The solid line denotes the unity line, where the ration of predicted to observed values equals 1. The dotted line indicates a two-fold error margin. The green triangle represents data of K_p_ from report; The red square represents of data K_p_ from QSAR prediction; The orange dot represents data of K_p_ from interspecies extrapolation.

The observed *versus* predicted plasma concentration and time profiles after intravenous administration are plotted in Figure 3. Based on visual inspection, improved concordance between observed data from the report and predicted data constructed using K_p_ reported in the research in rat model, as well as between the observed data from the report and predicted data developed utilizing QSAR predicted K_p_ in human model. The model parameters, clinical study information and pharmacokinetic parameters are listed Table 3. The observed and predicted values for V_ss_ and AUC_0-t_ are compared and illustrated in Figure 4. The predicted values for most pharmacokinetic parameters fell within a 2-fold error margin of the observed values. However, the V_ss_ derived from human models through interspecies extrapolation exceeded this range, although it remained within a 3-fold error margin.

**TABLE 3:** The model parameters, clinical study information and pharmacokinetic parameters of fentanyl in rats and humans.

Species	Rats			Humans		
Dose	1.75 μg			0.1 mg		
Gender	female	male		both	male	
Age	6–8 weeks			19–32 years	30 years	
Average body weight	0.25 kg			67.1 kg	70 kg	

	Observed	Predicted^1^	Predicted^2^	Observed	Predicted^3^	Predicted^4^
CL_sys_ (L/h)	0.493	0.493	0.493	62.66	59.324	59.324
V_ss_ (L)	1.496	1.246	1.811	364.9	757.847	333.525
T_1/2_ (h)	1.60	1.751	2.546	N/D	8.853	3.896
AUC_0-t_ (pg·h/mL)	2,272	2,684	2,196	1530.6	1408.9	1590
AUC_0-inf_ (pg·h/mL)	3,552	3,408	3,385	1595.9	1621.6	1683.6

^a^ Predicted^1^K_p_ from report; Predicted^2&4^. K_p_ from QSAR; Predicted^3^ K_p_ from interspecies extrapolation; CL_sys_, clearance; V_ss_, steady-state volume of distribution; T_1/2_, half-life; AUC_0-t_ & AUC_0-inf_, the total area under the plasma curve from time t and infinity.

### 3.3 Human PBPK modeling of 34 fentanyl analogs derived from QSAR-predicted results

**FIGURE 5:**
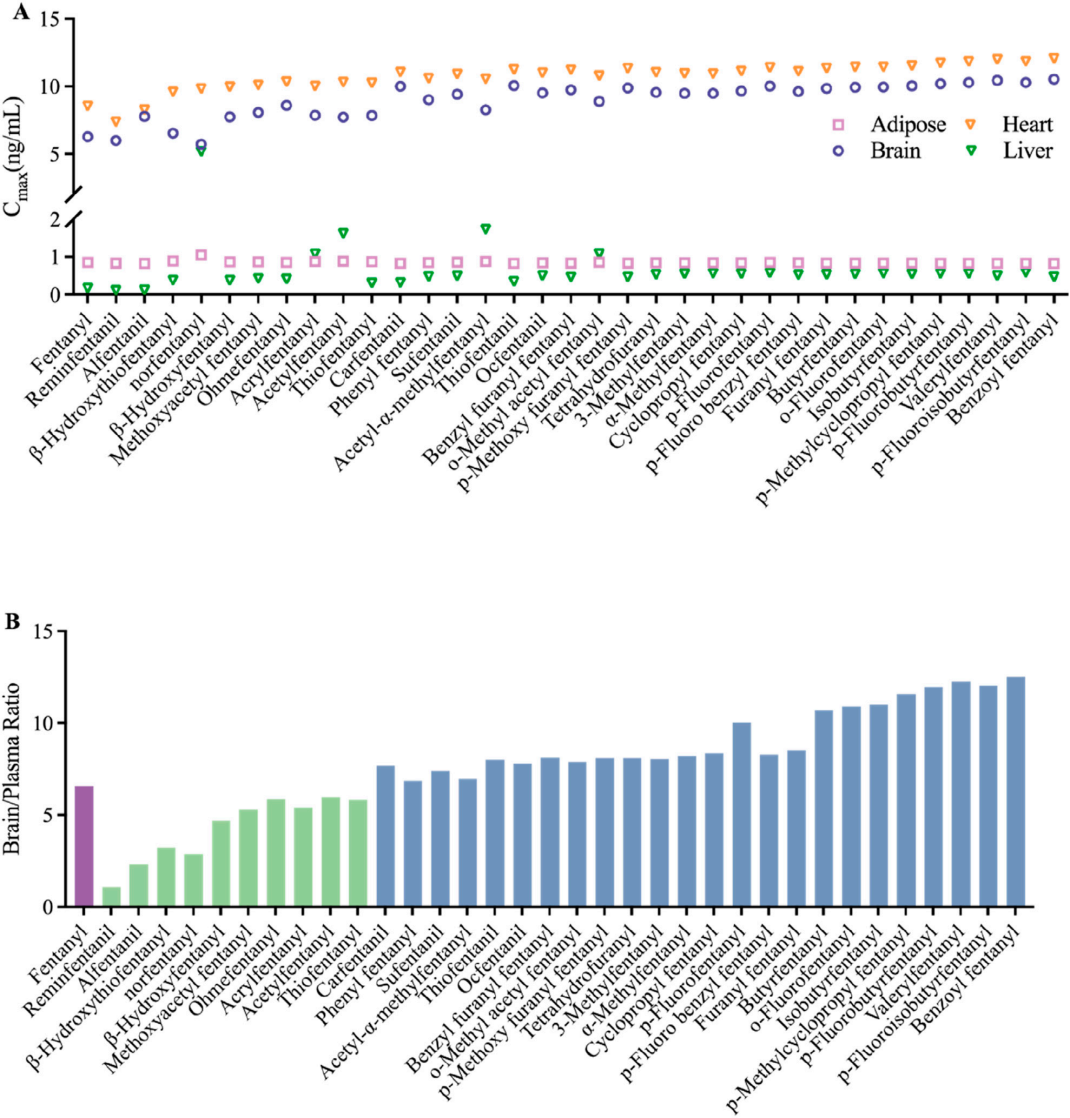
Predicted distribution in tissues of fentanyl analogs following intravenous administration **(A)** C_max_ of adipose, brain, heart and liver; **(B)** the ratio of brain and plasma, green bars indicate ratios lower than fentanyl, blue bars indicate ratios higher than fentanyl.

The PBPK model for 34 fentanyl analogs following intravenous administration in the human was developed using the K_p_ predicted by QSAR. The predicted model parameters and pharmacokinetic parameters predicted are listed in Table 4. When the AUC of the 34 fentanyl analogs were compared to plasma data for fentanyl in humans, all AUCs fell within a 1.3-fold range of one another. Figure 5 illustrates the C_max_ of fentanyl analogs in the brain, heart, adipose and liver, and brain/plasma ratio. The results showed that drug concentrations in the brain and heart were significantly higher than in plasma.

**TABLE 4:** The predicted model parameters and pharmacokinetic parameters of 34 fentanyl analogs.

Acetylfentanyl	Log P	S_w_ (mg/mL)	F_up_ (%)	R_bp_	P_eff_ (10^–4^ cm^a^s^-1^)
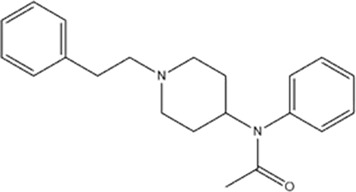	3.55	0.59	16.8	0.83	3.81
	CL_sys_ (L/h)	V_ss_ (L)	T_1/2_ (h)	AUC_0-t_ (pg·h/mL)	AUC_0-inf_ (pg·h/mL)
	62.66	250.140	2.766	1567	1595.9

Acetyl-α-methylfentanyl	Log P	S_w_ (mg/mL)	F_up_ (%)	R_bp_	P_eff_ (10^–4^ cm^a^s^-1^)
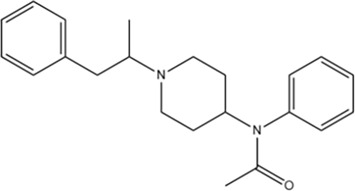	3.86	0.46	14.85	0.83	3.72
	CL_sys_ (L/h)	V_ss_ (L)	T_1/2_ (h)	AUC_0-t_ (pg·h/mL)	AUC_0-inf_ (pg·h/mL)
	62.66	308.742	3.415	1536.9	1595.4

Acrylfentanyl	Log P	S_w_ (mg/mL)	F_up_ (%)	R_bp_	P_eff_ (10^–4^ cm^a^s^-1^)
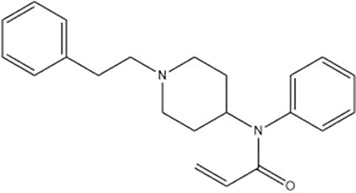	3.57	0.47	13.44	0.78	4.2
	CL_sys_ (L/h)	V_ss_ (L)	T_1/2_ (h)	AUC_0-t_ (pg·h/mL)	AUC_0-inf_ (pg·h/mL)
	59.393	400.880	4.94	1617.8	1772.2

Alfentanil	Log P	S_w_ (mg/mL)	F_up_ (%)	R_bp_	P_eff_ (10^–4^ cm^a^s^-1^)
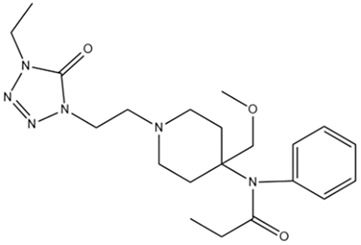	2.26	1.08	15.79	0.76	3.21
	CL_sys_ (L/h)	V_ss_ (L)	T_1/2_ (h)	AUC_0-t_ (pg·h/mL)	AUC_0-inf_ (pg·h/mL)
	53.931	72.644	0.933	1854.2	1854.2

Benzyl furanyl fentanyl	Log P	S_w_ (mg/mL)	F_up_ (%)	R_bp_	P_eff_ (10^–4^ cm^a^s^-1^)
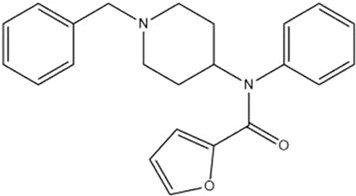	3.93	0.0763	5.88	0.76	4.41
	CL_sys_ (L/h)	V_ss_ (L)	T_1/2_ (h)	AUC_0-t_ (pg·h/mL)	AUC_0-inf_ (pg·h/mL)
	58.553	371.027	4.391	1582.2	1704.6

Benzyl fentanyl	Log P	S_w_ (mg/mL)	F_up_ (%)	R_bp_	P_eff_ (10^–4^ cm^a^s^-1^)
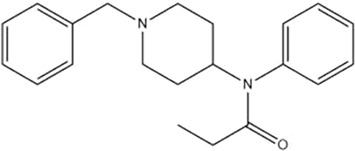	3.53	0.43	12.62	0.78	4.94
	CL_sys_ (L/h)	V_ss_ (L)	T_1/2_ (h)	AUC_0-t_ (pg·h/mL)	AUC_0-inf_ (pg·h/mL)
	60.094	288.717	3.329	1600.5	1663.4

Butyrfentanyl	Log P	S_w_ (mg/mL)	F_up_ (%)	R_bp_	P_eff_ (10^–4^ cm^a^s^-1^)
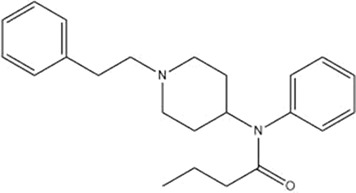	4.49	0.25	9.61	0.76	4.26
	CL_sys_ (L/h)	V_ss_ (L)	T_1/2_ (h)	AUC_0-t_ (pg·h/mL)	AUC_0-inf_ (pg·h/mL)
	58.553	492.190	5.825	1515.1	1694.8

Carfentanil	Log P	S_w_ (mg/mL)	F_up_ (%)	R_bp_	P_eff_ (10^–4^ cm^a^s^-1^)
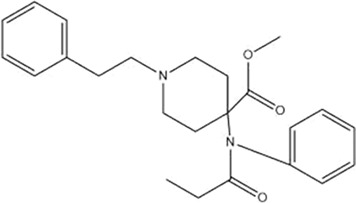	3.79	0.33	8.99	0.71	3.22
	CL_sys_ (L/h)	V_ss_ (L)	T_1/2_ (h)	AUC_0-t_ (pg·h/mL)	AUC_0-inf_ (pg·h/mL)
	54.701	286.141	3.625	1733.4	1827.1

Cyclopropyl fentanyl	Log P	S_w_ (mg/mL)	F_up_ (%)	R_bp_	P_eff_ (10^–4^ cm^a^s^-1^)
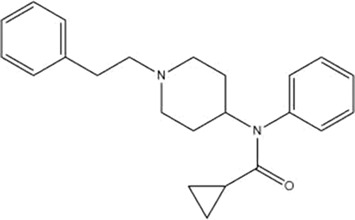	4.33	0.2	9.08	0.77	4.05
	CL_sys_ (L/h)	V_ss_ (L)	T_1/2_ (h)	AUC_0-t_ (pg·h/mL)	AUC_0-inf_ (pg·h/mL)
	59.324	444.197	5.189	1524.2	1677.6

Furanyl fentanyl	Log P	S_w_ (mg/mL)	F_up_ (%)	R_bp_	P_eff_ (10^–4^ cm^a^s^-1^)
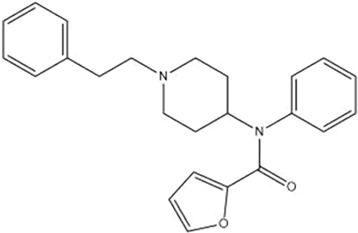	4.38	0.0861	6.46	0.76	3.31
	CL_sys_ (L/h)	V_ss_ (L)	T_1/2_ (h)	AUC_0-t_ (pg·h/mL)	AUC_0-inf_ (pg·h/mL)
	58.553	462.728	5.477	1658.9	1683.6

Isobutyrfentanyl	Log P	S_w_ (mg/mL)	F_up_ (%)	R_bp_	P_eff_ (10^–4^ cm^a^s^-1^)
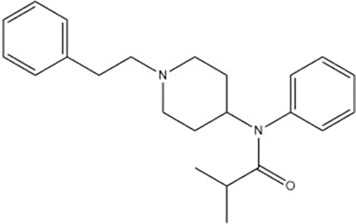	4.49	0.24	9.85	0.76	4.32
	CL_sys_ (L/h)	V_ss_ (L)	T_1/2_ (h)	AUC_0-t_ (pg·h/mL)	AUC_0-inf_ (pg·h/mL)
	58.553	511.739	6.057	1505.5	1692.6

Methoxyacetyl fentanyl	Log P	S_w_ (mg/mL)	F_up_ (%)	R_bp_	P_eff_ (10^–4^ cm^a^s^-1^)
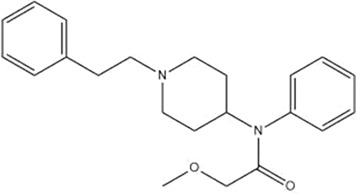	3.38	0.84	16.77	0.76	3.03
	CL_sys_ (L/h)	V_ss_ (L)	T_1/2_ (h)	AUC_0-t_ (pg·h/mL)	AUC_0-inf_ (pg·h/mL)
	58.553	199.652	2.363	1688.4	1707.8

Norfentanyl	Log P	S_w_ (mg/mL)	F_up_ (%)	R_bp_	P_eff_ (10^–4^ cm^a^s^-1^)
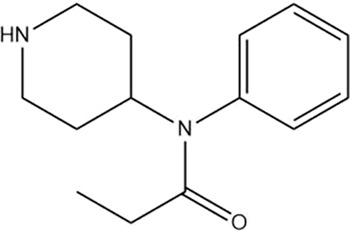	2.05	8.07	62.38	0.97	4.04
	CL_sys_ (L/h)	V_ss_ (L)	T_1/2_ (h)	AUC_0-t_ (pg·h/mL)	AUC_0-inf_ (pg·h/mL)
	62.66	146.271	1.618	1595.8	1593.7

Ocfentanil	Log P	S_w_ (mg/mL)	F_up_ (%)	R_bp_	P_eff_ (10^–4^ cm^a^s^-1^)
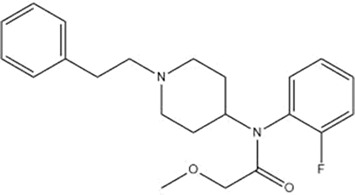	3.97	0.46	11.32	0.76	4.02
	CL_sys_ (L/h)	V_ss_ (L)	T_1/2_ (h)	AUC_0-t_ (pg·h/mL)	AUC_0-inf_ (pg·h/mL)
	58.553	364.286	4.311	1588.1	1704.6

o-Fluorofentanyl	Log P	S_w_ (mg/mL)	F_up_ (%)	R_bp_	P_eff_ (10^–4^ cm^a^s^-1^)
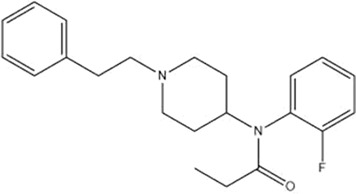	4.48	0.18	9.13	0.77	3.89
	CL_sys_ (L/h)	V_ss_ (L)	T_1/2_ (h)	AUC_0-t_ (pg·h/mL)	AUC_0-inf_ (pg·h/mL)
	59.324	513.51	5.999	1488.6	1670.8

Ohmefentanyl	Log P	S_w_ (mg/mL)	F_up_ (%)	R_bp_	P_eff_ (10^–4^ cm^a^s^-1^)
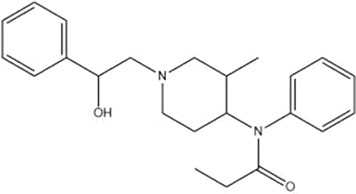	3.23	0.76	13.66	0.76	3.21
	CL_sys_ (L/h)	V_ss_ (L)	T_1/2_ (h)	AUC_0-t_ (pg·h/mL)	AUC_0-inf_ (pg·h/mL)
	58.553	215.207	2.547	1679.9	1707.8

o-Methyl acetyl fentanyl	Log P	S_w_ (mg/mL)	F_up_ (%)	R_bp_	P_eff_ (10^–4^ cm^a^s^-1^)
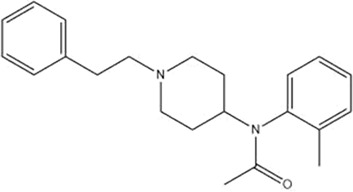	4.04	0.31	12.27	0.82	3.84
	CL_sys_ (L/h)	V_ss_ (L)	T_1/2_ (h)	AUC_0-t_ (pg·h/mL)	AUC_0-inf_ (pg·h/mL)
	62.66	380.079	4.204	1494.1	1593.2

p-Fluoro benzyl fentanyl	Log P	S_w_ (mg/mL)	F_up_ (%)	R_bp_	P_eff_ (10^–4^ cm^a^s^-1^)
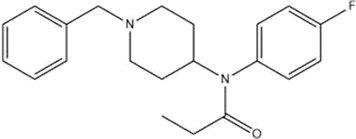	3.89	0.24	10.65	0.78	4.8
	CL_sys_ (L/h)	V_ss_ (L)	T_1/2_ (h)	AUC_0-t_ (pg·h/mL)	AUC_0-inf_ (pg·h/mL)
	60.094	394.155	4.545	1536.1	1659.9

p-Fluorobutyrfentanyl	Log P	S_w_ (mg/mL)	F_up_ (%)	R_bp_	P_eff_ (10^–4^ cm^a^s^-1^)
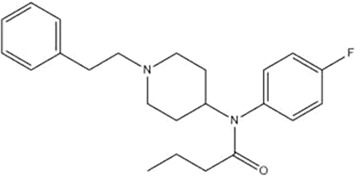	4.88	0.15	8.5	0.76	4.02
	CL_sys_ (L/h)	V_ss_ (L)	T_1/2_ (h)	AUC_0-t_ (pg·h/mL)	AUC_0-inf_ (pg·h/mL)
	58.553	623.339	7.377	1457.6	1676.1

p-Fluorofentanyl	Log P	S_w_ (mg/mL)	F_up_ (%)	R_bp_	P_eff_ (10^–4^ cm^a^s^-1^)
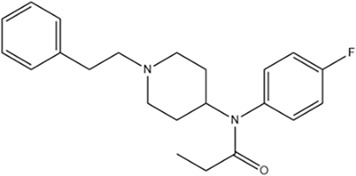	4.37	0.2	10.58	0.77	3.84
	CL_sys_ (L/h)	V_ss_ (L)	T_1/2_ (h)	AUC_0-t_ (pg·h/mL)	AUC_0-inf_ (pg·h/mL)
	59.324	460.433	5.379	1514.7	1675.5

p-Fluoroisobutyrfentanyl	Log P	S_w_ (mg/mL)	F_up_ (%)	R_bp_	P_eff_ (10^–4^ cm^a^s^-1^)
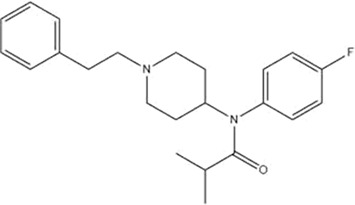	4.89	0.14	8.62	0.77	4.07
	CL_sys_ (L/h)	V_ss_ (L)	T_1/2_ (h)	AUC_0-t_ (pg·h/mL)	AUC_0-inf_ (pg·h/mL)
	59.324	644.489	7.529	1434	1651.2

Phenyl fentanyl	Log P	S_w_ (mg/mL)	F_up_ (%)	R_bp_	P_eff_ (10^–4^ cm^a^s^-1^)
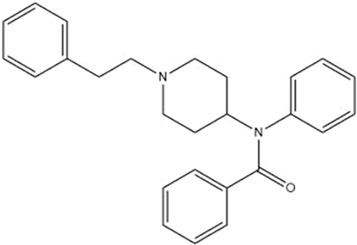	5.04	0.0494	4.94	0.73	4.63
	CL_sys_ (L/h)	V_ss_ (L)	T_1/2_ (h)	AUC_0-t_ (pg·h/mL)	AUC_0-inf_ (pg·h/mL)
	56.242	633.984	7.812	1503.5	1742.3

p-Methoxy furanyl fentanyl	Log P	S_w_ (mg/mL)	F_up_ (%)	R_bp_	P_eff_ (10^–4^ cm^a^s^-1^)
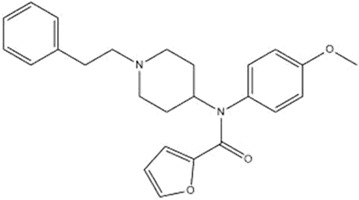	4.26	0.11	7.14	0.73	2.68
	CL_sys_ (L/h)	V_ss_ (L)	T_1/2_ (h)	AUC_0-t_ (pg·h/mL)	AUC_0-inf_ (pg·h/mL)
	56.242	400.880	4.94	1617.8	1772.2

p-Methylcyclopropyl fentanyl	Log P	S_w_ (mg/mL)	F_up_ (%)	R_bp_	P_eff_ (10^–4^ cm^a^s^-1^)
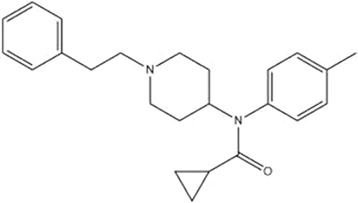	4.79	0.11	8.05	0.97	3.99
	CL_sys_ (L/h)	V_ss_ (L)	T_1/2_ (h)	AUC_0-t_ (pg·h/mL)	AUC_0-inf_ (pg·h/mL)
	58.553	587.442	6.953	1471.7	1682.1

Remifentanil	Log P	S_w_ (mg/mL)	F_up_ (%)	R_bp_	P_eff_ (10^–4^ cm^a^s^-1^)
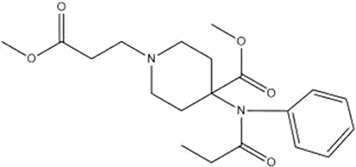	1.95	1.13	26.57	0.7	3.68
	CL_sys_ (L/h)	V_ss_ (L)	T_1/2_ (h)	AUC_0-t_ (pg·h/mL)	AUC_0-inf_ (pg·h/mL)
	58.553	48.262	0.571	1707.8	1707.8

Sufentanil	Log P	S_w_ (mg/mL)	F_up_ (%)	R_bp_	P_eff_ (10^–4^ cm^a^s^-1^)
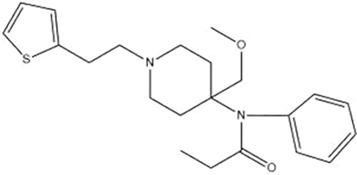	3.85	0.22	8.91	0.76	3.91
	CL_sys_ (L/h)	V_ss_ (L)	T_1/2_ (h)	AUC_0-t_ (pg·h/mL)	AUC_0-inf_ (pg·h/mL)
	58.553	308.601	3.652	1622.2	1706.5

Tetrahydrofuranyl fentanyl	Log P	S_w_ (mg/mL)	F_up_ (%)	R_bp_	P_eff_ (10^–4^ cm^a^s^-1^)
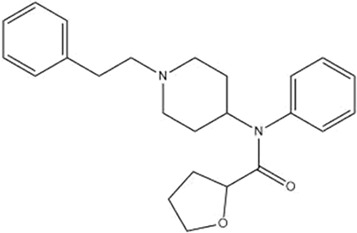	4.11	0.39	9.94	0.77	3.18
	CL_sys_ (L/h)	V_ss_ (L)	T_1/2_ (h)	AUC_0-t_ (pg·h/mL)	AUC_0-inf_ (pg·h/mL)
	59.324	401.909	4.695	1548.3	1680.7

Thiofentanil	Log P	S_w_ (mg/mL)	F_up_ (%)	R_bp_	P_eff_ (10^–4^ cm^a^s^-1^)
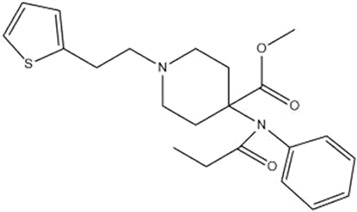	3.4	0.078	9.39	0.73	4.46
	CL_sys_ (L/h)	V_ss_ (L)	T_1/2_ (h)	AUC_0-t_ (pg·h/mL)	AUC_0-inf_ (pg·h/mL)
	56.242	319.869	3.941	1669.4	1776.4

Thiofentanyl	Log P	S_w_ (mg/mL)	F_up_ (%)	R_bp_	P_eff_ (10^–4^ cm^a^s^-1^)
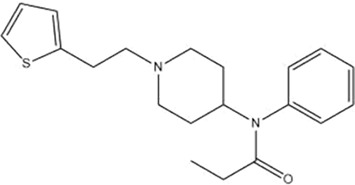	3.65	0.16	11.89	0.81	4.17
	CL_sys_ (L/h)	V_ss_ (L)	T_1/2_ (h)	AUC_0-t_ (pg·h/mL)	AUC_0-inf_ (pg·h/mL)
	62.66	252.001	2.787	1564.3	1595.8

Valerylfentanyl	Log P	S_w_ (mg/mL)	F_up_ (%)	R_bp_	P_eff_ (10^–4^ cm^a^s^-1^)
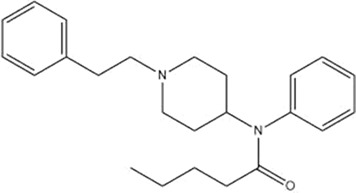	4.96	0.16	7.96	0.74	4.27
	CL_sys_ (L/h)	V_ss_ (L)	T_1/2_ (h)	AUC_0-t_ (pg·h/mL)	AUC_0-inf_ (pg·h/mL)
	57.035	624.455	7.587	1489.5	1719.9

α-Methylfentanyl	Log P	S_w_ (mg/mL)	F_up_ (%)	R_bp_	P_eff_ (10^–4^ cm^a^s^-1^)
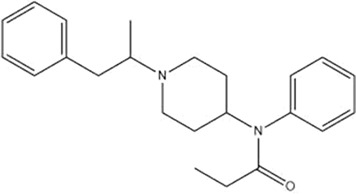	4.3	0.27	11.19	0.78	3.98
	CL_sys_ (L/h)	V_ss_ (L)	T_1/2_ (h)	AUC_0-t_ (pg·h/mL)	AUC_0-inf_ (pg·h/mL)
	60.094	428.159	4.937	1517.2	1657.6

β-Hydroxyfentanyl	Log P	S_w_ (mg/mL)	F_up_ (%)	R_bp_	P_eff_ (10^–4^ cm^a^s^-1^)
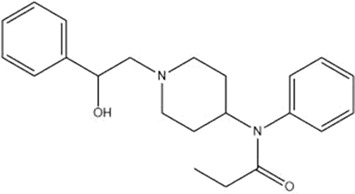	2.92	0.96	14.87	0.77	3.29
	CL_sys_ (L/h)	V_ss_ (L)	T_1/2_ (h)	AUC_0-t_ (pg·h/mL)	AUC_0-inf_ (pg·h/mL)
	59.324	166.633	1.947	1678	1685.7

β-Hydroxythiofentanyl	Log P	S_w_ (mg/mL)	F_up_ (%)	R_bp_	P_eff_ (10^–4^ cm^a^s^-1^)
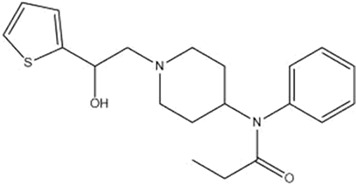	2.59	0.48	14.05	0.8	3.35
	CL_sys_ (L/h)	V_ss_ (L)	T_1/2_ (h)	AUC_0-t_ (pg·h/mL)	AUC_0-inf_ (pg·h/mL)
	61.635	132.097	1.485	1621.2	1622.5

3-Methyl fentanyl	Log P	S_w_ (mg/mL)	F_up_ (%)	R_bp_	P_eff_ (10^–4^ cm^a^s^-1^)
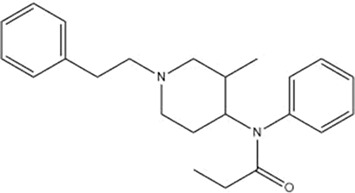	4.36	0.27	10.37	0.77	3.99
	CL_sys_ (L/h)	V_ss_ (L)	T_1/2_ (h)	AUC_0-t_ (pg·h/mL)	AUC_0-inf_ (pg·h/mL)
	59.324	419.576	4.901	1538.1	1679.4

^a^ Alphabetical order.

## 4 Discussion

### 4.1 Assessment of the viability of the modeling approach

An important factor that hinders the application of PBPK models in drug research is the substantial amount of data required for model construction. This encompasses the experimental determination of tissue affinities, particularly K_p_, which can be both costly and time-consuming. The most recent research on the modeling of PBPK for fentanyl and its analogs employs K_p_ derived from mathematical formulas or assumes that the same data applies to both humans and rats (Han et al., 2025; Rodgers et al., 2005). In addition, the extrapolation of animal data to humans is a widely accepted method in PBPK modeling. One study has successfully developed a PBPK model for cocaine and its metabolite benzoylcholine in humans based on extrapolated data from rats (Bravo-Gómez et al., 2019). Therefore, our study employed experimental measurements, QSAR predictions, and interspecies extrapolation to derive K_p_ for modeling purposes. To verify the applicability of the QSAR prediction modeling method for K_p_ to fentanyl analogs, rather than being restricted solely to fentanyl, our study employed this approach to develop a rat PBPK model of β-hydroxythiofentanyl, then evaluated the model against experimentally determined drug-time profiles and pharmacokinetic parameters. By comparing several fentanyl models that we have developed, it is observed that the experimentally determined K_p_ is preferred in the rat model, whereas the QSAR-predicted K_p_ is favored in the human model. The results demonstrated that the model exhibited high predictive accuracy and confirmed the feasibility of this approach. Eventually, our research constructed 34 fentanyl human PBPK models for fentanyl analogs following this method.

### 4.2 Analysis of model results

#### 4.2.1 Comparison of data on fentanyl analogs with existing studies

Intravenous fentanyl and Sufentanil are primarily utilized for general anesthesia, while alfentanil and remifentanil serve the purposes of analgesia and sedation. Notably, remifentanil is particularly well-suited for short-term or outpatient procedures (Ziesenitz et al., 2018). Given the specific medical applications of these three fentanyls, their pharmacokinetics have been extensively investigated in existing studies. In contrast, there is a significant lack of experimental data regarding other illicitly manufactured fentanyl analogs in both animal models and human volunteers. Consequently, we selected alfentanil, Sufentanil, and remifentanil as representative drugs to evaluate the predictive accuracy of our developed model using PK parameters documented in the current research (details shown in Table 5).

**TABLE 5:** The reported observed and predicted values of selected PK (logP and F_up_) and QSAR (CL and T_1/2_) parameters for Sufentanil, Alfentanil, and Remifentanil.

Analogs	Source	Route	Dose (μg/kg)	Gender	Average age (years)	Average weight (kg)	logP^*^	F_up_ (%)	CL (L/h)	T_1/2_(h)	References
Sufentanil	Observed	Intravenous	5	3F 7M	45.5	71.1	3.95	7.5	54.01	3.1	Bovill et al. (1984)
	Predicted	Intravenous	7	M	30	70	3.85	8.91	58.55	3.652	
Alfentanil	Observed	Intravenous	2.4	2F 5M	39.9	70.3	2.16	12.75	31.44	1.21	Bower and Hull (1982)
	Predicted	Intravenous	7	M	30	70	2.26	15.79	53.931	0.93	
Remifentanil	Observed	Intravenous	5	both	38.2	79.2	1.4	N/D	184.5	0.29	Westmoreland et al. (1993)
	Predicted	Intravenous	7	M	30	70	1.95	26.57	58.553	0.571	

^*^ The observed value of logP is provided by PubChem.

In a study on sufentanil, the pharmacokinetic characteristics of 10 surgical patients who received an intravenous dose of 5 μg/kg sufentanil were reported. The results showed a mean half-life (T_1/2_) of 164 ± 22 min (converted to hours: 2.73 ± 0.37 h) and a mean steady-state volume of distribution (Vdss) of 1.7 ± 0.2 L/kg (Bovill et al., 1984). Based on a standard body weight of 70 kg, the absolute Vdss value in this study was calculated as 119 ± 14 L. The T_1/2_ of sufentanil predicted in this study was 3.652 h, with an error ratio of 1.34 (3.652/2.73) relative to the literature value, within the acceptable error range of 1.3–1.7 fold. The predicted steady-state volume of distribution (V_ss_) was 308.601 L, resulting in an error ratio of approximately 2.59 when compared to the literature-derived absolute value (119 ± 14 L). This discrepancy may be attributed to two main factors: first, the standardized setting of human physiological parameters in the model (e.g., uniform 70 kg body weight, standard tissue blood perfusion rates) *versus* inherent inter-individual variability in patient physiology (e.g., age, body weight distribution, organ function status); second, differences between the experimental measurement method for Vdss in the literature and the model’s prediction algorithm (derived from QSAR-predicted tissue partition coefficients, Kp). Despite the numerical difference, both values reflect the large volume of distribution of sufentanil, indicating its extensive tissue distribution *in vivo*, which confirms consistency in qualitative trends.

For alfentanil, a study on healthy volunteers reported that following intravenous administration of 170 μg alfentanil (equivalent to approximately 2.4 μg/kg for a 70 kg individual), the mean T_1/2_ was 1.21 h (Bower and Hull, 1982). The T_1/2_ of alfentanil predicted in this study was 0.93 h, with an error ratio of 1.75 (1.63/0.93) relative to the literature value—approaching the upper limit of the acceptable 1.7-fold error range. The predicted systemic clearance (CL_sys_) was 53.931 L/h. Although no direct measurement of alfentanil clearance was reported in the literature, clearance was estimated using the relationship between AUC_0-t_ (area under the plasma concentration-time curve) and dose (CL = dose/AUC), yielding an estimated clearance of approximately 31.44 L/h (Bower and Hull, 1982). The error ratio between the predicted and estimated clearance values was 1.71 (53.931/31.44), which also fell within the acceptable range. Both values consistently indicate the relatively rapid clearance of alfentanil.

In a study on remifentanil, surgical patients received intravenous doses of 2, 5, 15, and 30 μg/kg remifentanil, and blood samples were analyzed. The results showed that T_1/2_ increased with dose, measuring 10.19, 14.35, 15.67, and 20.47 min (converted to hours: 0.17, 0.24, 0.26, and 0.34 h), respectively (Westmoreland et al., 1993). The T_1/2_ of remifentanil predicted in this study was 0.571 h, with error ratios ranging from 1.68 to 3.36 (0.571/0.34 to 0.571/0.17) relative to the literature values for each dose group. It is important to note that the subjects in this literature study were surgical patients, and remifentanil—an ultra-short-acting opioid—undergoes metabolism that is significantly influenced by esterase activity, hepatic/renal function, and surgical stress. In contrast, the model in this study was constructed based on physiological parameters of healthy individuals and did not account for the effects of pathological conditions or stress responses on metabolic enzyme activity, which likely contributed to the larger discrepancy between predicted and literature values. Additionally, regarding the AUC and Cmax (peak plasma concentration) of remifentanil, only one 2004 study reporting data from ICU patients with renal dysfunction was identified (Pitsiu et al., 2004). These patients had impaired renal function; although remifentanil is primarily metabolized by non-specific esterases (with minimal impact from renal function), the overall physiological state of the patients (e.g., circulatory stability, metabolic enzyme expression levels) differed significantly from that of healthy individuals. This made the data poorly comparable to the predictions of the healthy human-based model in this study, so these parameters were not included in quantitative comparisons.

Beyond the three aforementioned drugs, recent literature has reported pharmacokinetic data for other fentanyl analogs, but most focus on non-intravenous administration routes (e.g., sublingual, subcutaneous implantation). For example, one study (van de Donk et al., 2018) reported the bioavailability and AUC of sublingual fentanyl wafers; however, this route involves drug absorption through the oral mucosa, which differs significantly from the intravenous route (no first-pass effect, simple absorption process) used in this study in terms of pharmacokinetic characteristics. Another study measured the PK parameters of analogs such as acetylfentanyl and butyrylfentanyl *via* subcutaneous injection (Canfield and Sprague, 2024), which involves slow drug diffusion and absorption in subcutaneous tissue—leading to differences in the timing of peak plasma concentration and AUC calculation methods compared to intravenous injection. These differences prevent direct quantitative comparison with the predictions of this study. Therefore, such literature data from non-intravenous routes were not included in the comparative analysis.

To further verify the reliability of the model’s input parameters, this study supplemented a comparison between QSAR-predicted key physicochemical properties (e.g., logP, F_up_) and experimentally measured values. As shown in Table 5: 1) For sufentanil, the experimental logP value was 3.95, and the QSAR-predicted value was 3.85, with an error of only 2.5%. 2) For alfentanil, the experimental logP value was 2.16, and the QSAR-predicted value was 2.26, with an error of 4.6%. 3) For remifentanil, the experimental logP value was 1.4, and the QSAR-predicted value was 1.95, with an error of 39.3% (primarily due to the presence of ester and morpholine groups in the remifentanil molecule, which cause variability in experimentally measured logP values across different solvent systems).

In terms of Fup: 1) The experimental Fup value for sufentanil was 7.5%, and the QSAR-predicted value was 8.91%, with an error of 18.8%. 2) The experimental Fup value for alfentanil was 12.75%, and the QSAR-predicted value was 15.79%, with an error of 23.8%.

Overall, the QSAR-predicted values for logP (except for remifentanil) and Fup showed minimal deviation from experimental values, particularly for logP, which exhibited high predictive accuracy. As a key parameter influencing drug tissue partition coefficients (Kp) and transmembrane transport capacity, the accurate prediction of logP directly ensures the reliability of the PBPK model’s simulations of drug distribution and clearance processes. Even for remifentanil—where logP prediction showed a larger deviation—the QSAR-predicted Fup value (26.57%) was consistent with the qualitative characteristic of “low plasma protein binding of remifentanil” reported in the literature, still providing a reasonable basis for input parameters in the model.

In summary, the errors between the predicted PK parameters (T_1_/_2_, CLsys) of sufentanil, alfentanil, and remifentanil in this study and the literature experimental values mostly fell within the 1.3–1.7 fold range. Additionally, the QSAR-predicted values of key physicochemical properties showed minimal deviation from experimental values, indicating high predictive accuracy of the model. For parameters with moderate deviations (e.g., V_ss_ of sufentanil), the causes can be reasonably explained by factors such as differences in administration routes, standardized physiological parameter settings, and experimental conditions—with consistent qualitative trends with the literature. This result significantly enhances the credibility of the PK predictions for other fentanyl analogs lacking experimental data in this study, providing a reliable model basis for subsequent assessments of the abuse potential of these analogs (e.g., evaluating central nervous system penetration based on brain/plasma concentration ratios).

#### 4.2.2 Impact of the structural characteristics of fentanyl analogs on pharmacokinetics

New psychoactive substances (NPS) are frequently synthesized in clandestine laboratories with the intention of chemically modifying controlled drugs to circumvent legal regulations. Illegally manufactured substances resembling fentanyl are among the contributing factors to the global rise in fatalities associated with the NPS. Fentanyl analogs are frequently marketed as fentanyl itself, offered as substitutes for other substances, or incorporated into counterfeit prescription medications (Armenian et al., 2018). Due to the insufficient PK and PD evaluation of these analogs, potential users and others who may be exposed to such drugs remain unaware of their effects, hazards, and potency. Our study aimed to predict the PK of 34 fentanyl analogs and sought to establish a relationship between their structural characteristics and pharmacokinetic profiles. A hypothesis presented in the research suggests that pharmacokinetic parameters increase as fentanyl analogs become more lipophilic. The pharmacokinetics of acetylfentanyl, butyrylfentanyl, cyclopropylfentanyl, and valerylfentanyl were evaluated in rats following s. c injection at a dosage of 300 μg/kg. The results indicated that acetylfentanyl exhibited the shortest carbon side chain along with the lowest T_1/2_ and C_max_ (Canfield and Sprague, 2024). It has been proposed that the incorporation of the functional group 3-carbomethoxy into fentanyl analogs may have the potential to reduce the duration of action by modifying their pharmacokinetic properties. This is attributed to the fact that more hydrophilic groups tend to accumulate minimally, if at all, in adipose tissue and are rapidly excreted. Additionally, it may be due to the susceptibility of the carbomethoxy group to rapid hydrolysis by non-specific esterases (Vucković et al., 2009). Therefore, it can be concluded that the physicochemical property exerting a significant influence on the pharmacokinetics of fentanyl analogs is lipophilicity. In conjunction with the predictive results, we summarize the relationship between the structural characteristics of fentanyl analogs and their pharmacokinetic profiles.

• Influence of carbon side chain on the *in vivo* pharmacokinetic characteristics of fentanyls

1. Pharmacokinetic parameters, including T_1/2_ and V_ss_, exhibit an increase with longer carbon side chain lengths, while AUC demonstrates a decrease as carbon side chain length increases.
2. Functional groups present on the carbon side chain, such as methoxyacetyl, are associated with a reduction in both T_1/2_ and V_ss_. In contrast, the presence of furanyl, tetrahydrofuranyl, cyclopropyl, and phenyl groups tends to increase both T_1/2_ and V_ss_ to varying extents. The order is tetrahydrofuranyl < cyclopropyl < furanyl < phenyl.

• Influence of the position 3, or four of the piperidine ring on the *in vivo* pharmacokinetic characteristics of fentanyls

1. Both 3-carbomethoxy and methoxymethyl substituents at the four-position of the piperidine ring are associated with a reduction in T_1/2_, whereas a methyl group at the three-position is linked to an increase in T_1/2_.

• Influence of the directly connected to the nitrogen on the vivo pharmacokinetic characteristics of fentanyls.

1. The introduction of hydroxyl group to the carbon chain that is directly bonded to the nitrogen results in a reduction of both the T_1/2_ and V_ss_. Conversely, the incorporation of methyl group leads to an enhancement in these two parameters.
2. The incorporation of functional groups such as thienyl, methyl and phenyl can decrease the T_1/2_ and V_ss_ to varying extents. The order is phenyl < thienyl < methyl.

#### 4.2.3 Distribution of fentanyl analogs in tissues and organs

The brain-blood ratio serves as a crucial metric for estimating the pharmacokinetics of the central nervous system (CNS). Among various methodologies, the brain-blood ratio is favored over more complex techniques such as *in situ* brain perfusion and microdialysis due to its simplicity and practicality. Compounds exhibiting a higher brain-blood ratio demonstrate an enhanced capacity to traverse the blood-brain barrier and exert effects on the CNS. For any given compound, a ratio that approaches or slightly exceeds one suggests that it can readily cross the blood-brain barrier, whereas a significantly elevated ratio indicates a strong likelihood of accumulation within brain tissue (Kulkarni et al., 2016). A study has demonstrated that the brain-blood ratio for p-fluorofentanyl were significantly higher than that for fentanyl in several critical regions of the brain. This result indicates that the heightened toxicity associated with p-fluorofentanyl may be attributed to its enhanced permeability across the blood-brain barrier and increased exposure within brain tissue (Canfield and Sprague, 2025). This observation aligns with our prediction that the brain-blood ratio for p-fluorofentanyl is greater than that of fentanyl. The blue bars in Figure 5B highlight compounds exhibiting higher brain-blood ratios compared to fentanyl, which, as analogs of fentanyl, may possess a greater potential for abuse and pose increased risks relative to fentanyl. In addition, the use of opioids may be associated with several cardiovascular disorders, including cardiac arrest, tachycardia, bradycardia, and palpitations (Dai et al., 2024). The elevated C_max_ of fentanyl and its derivates observed in the heart, as depicted in Figure 5A, indicates a potential for cardiotoxicity.

### 4.3 Comparison with the existing PBPK reports on fentanyl analogs

High-throughput (HT)-PBPK modeling for fentanyl analogs is grounded in a growing body of domain-specific research, whose findings both support and constrain the present QSAR-PBPK framework.

Key insights from fentanyl PBPK literature align with our study’s design and results. Björkman et al. (Björkman, 2003) demonstrated that simplifying fentanyl’s PBPK model—while prioritizing core parameters (tissue/blood partition coefficient, Kp; plasma protein unbound fraction, Fup)—retains predictive accuracy, validating our focus on QSAR-derived Kp (a critical driver of tissue distribution). Notably, Björkman also highlighted that cross-species Kp extrapolation (rat-to-human) introduces ≥30% error for lipophilic opioids, which echoes our observation that QSAR-predicted human Kp reduced steady-state volume of distribution (V_ss_) error to <1.5-fold (vs. >3-fold for extrapolation, Section 3.2). Population-specific PBPK studies further contextualize our model’s scope. Alsmadi (Alsmadi, 2023) and Kovar et al. (Kovar et al., 2020) showed that age-dependent physiology (e.g., pediatric CYP3A4 activity, neonatal tissue perfusion) alters fentanyl PK predictions by 25%–40%. While our use of standardized adult parameters (70 kg, healthy physiology) enables reliable analog-to-analog comparisons, these studies confirm that population-specific adjustments would be required for clinical translation—a key limitation.

Literature on fentanyl PBPK also supports QSAR’s utility for parameterization. Ni et al. (Ni et al., 2024) used QSAR-predicted enzyme inhibition constants (Ki) to model fentanyl-ritonavir interactions, yielding AUC fold-changes within 1.2-fold of clinical data—reinforcing our use of QSAR for K_p_ and physicochemical properties (logP, solubility) when experimental data are scarce. In contrast, Shankaran et al. (Shankaran et al., 2013) showed non-intravenous fentanyl models require route-specific absorption parameters (e.g., nasal permeability) to avoid ≥2-fold error, justifying our focus on intravenous administration (minimizing absorption uncertainty).

For novel analogs, Canfield and Sprague (Canfield and Sprague, 2024) reported a strong correlation (*R* ^2^ = 0.89) between carbon side-chain length and T_1/2_ for illicit fentanyls—consistent with our predictions (e.g., valerylfentanyl, C5: T_1/2_ = 7.587 h; acetylfentanyl, C2: T_1/2_ = 2.766 h, Table 4). However, their observation that analogs with novel functional groups (e.g., furanyl) exhibit unexpected tissue binding underscores QSAR limitations for structurally divergent compounds.

### 4.4 Limitations of research

This study does have certain limitations. Firstly, given that fentanyl analogs represent an emerging class of controlled substances, clinical data are exceedingly scarce and cannot be utilized to evaluate the accuracy of our developed model. While our study proposes novel approaches for model assessment, the incorporation of additional clinical data would significantly enhance the validity of our model results. In addition, this study has established a PBPK model exclusively for fentanyl and its analogs following intravenous administration. However, other routes of administration, such as oral and nasal inhalation, have yet to be explored. To further investigate the pharmacokinetics of these substances, it is essential to develop additional routes of administration and conduct models after multiple administrations.

## 5 Conclusion

After a thorough evaluation of the modeling approach, QSAR-based PBPK models for 34 fentanyl analogs were developed. These models provide quantitative estimates of the pharmacokinetics of 34 fentanyl analogs in humans, with predictions for clinically validated analogs (e.g., sufentanil, alfentanil) aligning with available clinical data. These estimates help address critical data gaps in fentanyl analog hazard assessment and support preliminary prioritization of analogs for further experimental validation. It provided valuable insights that can not only guide further *in vivo* and *in vitro* experiments but also facilitate a preliminary assessment of their potential for abuse. This significantly addresses the existing data gap in this area.

## Data Availability

The original contributions presented in the study are publicly available. This data can be found here: https://data.mendeley.com/datasets/92jxfc48gg/1.
